# Changes in Mitochondrial Carriers Exhibit Stress-Specific Signatures in INS-1Eβ-Cells Exposed to Glucose Versus Fatty Acids

**DOI:** 10.1371/journal.pone.0082364

**Published:** 2013-12-12

**Authors:** Thierry Brun, Pasquale Scarcia, Ning Li, Pascale Gaudet, Dominique Duhamel, Ferdinando Palmieri, Pierre Maechler

**Affiliations:** 1 Department of Cell Physiology and Metabolism, University of Geneva, Medical Center, Geneva, Switzerland; 2 Department of Biosciences, Biotechnologies and Biopharmaceutics, University of Bari, Bari, Italy; 3 Center of Excellence in Comparative Genomics (CEGBA), University of Bari, Bari, Italy; 4 Swiss Institute of Bioinformatics (SIB) and University of Geneva, Medical Center, Geneva, Switzerland; University of Bremen, Germany

## Abstract

Chronic exposure of β-cells to metabolic stresses impairs their function and potentially induces apoptosis. Mitochondria play a central role in coupling glucose metabolism to insulin secretion. However, little is known on mitochondrial responses to specific stresses; *i.e*. low *versus* high glucose, saturated *versus* unsaturated fatty acids, or oxidative stress. INS-1E cells were exposed for 3 days to 5.6 mM glucose, 25 mM glucose, 0.4 mM palmitate, and 0.4 mM oleate. Culture at standard 11.1 mM glucose served as no-stress control and transient oxidative stress (200 µM H_2_O_2_ for 10 min at day 0) served as positive stressful condition. Mito-array analyzed transcripts of 60 mitochondrion-associated genes with special focus on members of the *Slc25* family. Transcripts of interest were evaluated at the protein level by immunoblotting. Bioinformatics analyzed the expression profiles to delineate comprehensive networks. Chronic exposure to the different metabolic stresses impaired glucose-stimulated insulin secretion; revealing glucotoxicity and lipo-dysfunction. Both saturated and unsaturated fatty acids increased expression of the carnitine/acylcarnitine carrier CAC, whereas the citrate carrier CIC and energy sensor SIRT1 were specifically upregulated by palmitate and oleate, respectively. High glucose upregulated CIC, the dicarboxylate carrier DIC and glutamate carrier GC1. Conversely, it reduced expression of energy sensors (AMPK, SIRT1, SIRT4), metabolic genes, transcription factor PDX1, and anti-apoptotic Bcl2. This was associated with caspase-3 cleavage and cell death. Expression levels of GC1 and SIRT4 exhibited positive and negative glucose dose-response, respectively. Expression profiles of energy sensors and mitochondrial carriers were selectively modified by the different conditions, exhibiting stress-specific signatures.

## Introduction

In pancreatic β-cells, mitochondria participate to glucose-stimulated insulin secretion (GSIS) by generating metabolic signals [1] and by replenishing the tricarboxylic acid cycle (TCA) of its intermediates [2]. Mitochondrial dysfunction impairs GSIS and may promote β-cell death [3]. Such defects are favored by chronic exposure to elevated concentrations of glucose and fatty acids [4]. In contrast to the acute potentiation of GSIS by fatty acids, prolonged incubation induces β-cell lipo-dysfunction characterized by elevated basal insulin release and impaired glucose response. In most studies, unsaturated fatty acids (e.g. oleate) do not affect cell viability [5]–[7], whereas saturated fatty acids (e.g. palmitate) may promote ER stress and apoptosis [8]–[10]. The chronic effects of palmitate on cell viability are inversely correlated with the concentration of serum in the culture medium, ranging from nontoxic [11], [12] to highly toxic [8]–[10], [12]. The cytotoxicity of saturated fatty acids also depends on the duration of exposure and concomitant high glucose concentrations [7]. The associated glucolipotoxicity concept proposes that high glucose and fatty acids induce pleiotropic alterations associated with diabetes and the metabolic syndrome. In this context, metabolic stresses could lead to β-cell dysfunction and apoptosis. The molecular basis of glucolipotoxicity is not clear, although it requires active nutrient metabolism; in turn altering lipid partitioning, production of reactive oxygen species (ROS), and mitochondrial dysfunction [13], [14]. Mitochondria are both a major source of ROS and the primary target of oxidative attacks [15], [16]. Then, mitochondrial defects and oxidative stress might contribute to the diabetic state [14], [17].

The present work aimed at identifying mitochondrial molecular targets of the main metabolic stresses using INS-1E insulinoma cells. These stresses include low and high glucose concentrations, saturated and unsaturated fatty acids, and transient oxidative stress. In this context, the mitochondrial carriers of the nuclear-encoded *Slc25* gene family are of particular interest since the transport of a variety of metabolites across the inner mitochondrial membrane is ensured by these solute carriers. Tissue distribution and molecular characterization of mitochondrial carriers have been partially documented, as well as their role in metabolic pathways [18], [19]. Recently, several diseases caused by modifications of their genes were reported in humans, including the carriers for citrate/isocitrate CIC (*SLC25A1*), carnitine/acylcarnitine CAC (*SLC25A20*), aspartate/glutamate AGC1 (*SLC25A12*), and glutamate GC1 (*SLC25A22*) [20]. Regarding β-cells, we reported that downregulation of GC1 or AGC1 reduces GSIS [21], [22]. Using pharmacological and siRNA approaches, similar conclusions were raised for CIC, 2-oxoglutarate OGC (*Slc25a11*), dicarboxylate DIC (*Slc25a10*), and inorganic phosphate PiC (*Slc25a3*) [23]–[26]. The present study provides the first gene expression profile of 22 mitochondrial carriers of the *Slc25* gene family in insulin-secreting cells, both in healthy and metabolically stressed cells.

## Materials and Methods

### Cell culture and treatments

INS-1E β-cells, originally cloned in our laboratory [27], [28] from the parental rat INS-1 cell line [29], were grown in RPMI-1640 medium at 11.1 mM glucose supplemented with 10 mM HEPES, 5% (vol./vol.) heat-inactivated fetal calf serum (FCS), 2 mM L-glutamine, 100 U/ml penicillin, 100 µg/ml streptomycin, 1 mM sodium pyruvate and 50 µM β-mercaptoethanol [28]. After 4 days of pre-culture in 24-well plates (Falcon, OmniLab, Mettmenstetten, Switzerland) or 78 cm^2^ Petri dishes coated with polyornithine (Sigma-Aldrich, St Louis, MO), cells were further maintained for 3 days at either 11.1 mM glucose (G11, control) or exposed to different glucose concentrations: low 5.6 mM (G5.6) and high 25 mM (G25). Cells were also treated with 0.4 mM palmitate (saturated fatty acid C16:0; Palm) or with 0.4 mM oleate (unsaturated fatty acid C18:1; Olea) in the presence of 0.5% BSA at G11 in RPMI-1640 medium supplemented with 5% FCS as detailed previously [5], [30]. Stock solutions of fatty acids (palmitate and oleate; Sigma-Aldrich) bound to BSA were adjusted to 10 mM fatty acids using 1.8 mM fatty acid-free BSA before storage at −20°C under nitrogen. As positive stressful condition, INS-1E cells were exposed to a single transient oxidative stress (200 µM H_2_O_2_ for 10 min) at the end of the pre-culture period according to the dose and time course established previously [16]. INS-1E cells exposure to each kind of stresses was performed strictly in parallel.

### Insulin secretion assay, protein and DNA measurements

Insulin secretion assay was performed over a 30 min stimulation period as detailed previously [28]. Total cellular insulin content and secreted insulin were quantified by radioimmunoassay (Linco Research Inc., St. Charles, MO) using rat insulin as standard. Levels of protein were determined by Bradford assay (Pierce, Rockford, IL). DNA quantification was determined by UV absorbance at 460 nm using Hoechst 33258 (Molecular Probes, Eugene, OR) as fluorochrome. DNA extracted from INS-1E cells were used to establish the standard curves. Fluorescence measurements were monitored in a fluorometer (Fluostar Optima, BMG Technologies, Offenburg, Germany).

### Apoptosis measurements

INS-1E cells were seeded (10^4^ cells/well) in 24-well plates and treated as described above. Apoptosis was quantified using the Cell Death Detection ELISA^PLUS^ kit (Roche Diagnostics, Basel, Switzerland) according to the manufacturer's instructions. Alternatively, cell death was estimated by CASPASE 3 cleavage activation by immunoblotting (see below).

### Mito-array using TaqMan Micro Fluidic Cards

We designed a molecular screening array (Mito-array) based on pre-loaded micro fluidic card Real Time PCR system, allowing analysis of 60 selected genes. Two independent Mito-array experiments with the same culture conditions were performed and for each experiment, each culture condition was done in triplicate. For this experimental design, statistics have been performed on the mean values obtained from the triplicates for each independent experiment. INS-1E cells were cultured (3.0×10^6^ cells/dish) for 4 days before exposure to the various stress conditions for 3 days. Then, total RNA was extracted with TRIzol reagent (Invitrogen), treated with DNAse at 37°C for 30 min, and 5 µg cDNA of each sample was synthetized using Gene Amp kit (Applied Biosystem, Life Technologies Corporation, CA). Gene expression analysis was performed by pre-loaded TaqMan Low Density Array (LDA). Samples were analyzed using the ABI Prism 7900HT Real time PCR system (Applied Biosystems). Each line of the LDAs was loaded with 100 ng single-stranded cDNA and TaqMan Gene Expression Master Mix (Applied Biosystems) before cycling (50°C for 2 min, 94°C for 10 min, 97°C for 30 sec, and 59.7°C for 1 min). The 18S gene was used as stable housekeeping gene; 18S Ct values showing no statistic differences between conditions (Ct mean of all samples was 13.356 with SD = 0.8917). Quantification of relative gene expression was performed according to the comparative method 2^−ΔΔCt^ [31], with the ΔCt of INS-1E cells cultured at G11 as reference values. In the comparative method 2^−ΔΔCt^ = 2^−[ΔCt(sample) −ΔCt(calibrator)]^, where ΔCt (sample) = Ct (sample) − Ct (reference gene) and Ct stands for the threshold cycle, i.e. the PCR cycle number at which emitted fluorescence exceeds 10 times the SD of baseline emissions. For the reference value, ΔΔCt = 0 and 2^0^ = 1. For the cells grown under different culture conditions, the value of 2^−ΔΔCt^ indicates the fold change in mRNA values relative to G11 values (calibrator) normalized to 18S.

### Immunoblotting

After the culture (3.0×10^6^ cells/dish) and stress period of 3 days, protein extracts from total INS-1E cells and isolated mitochondria were prepared as described [16]. Proteins from total cells (18 µg/lane) and mitochondria (9 µg/lane) were separated by 10–12% SDS-PAGE before transfer onto polyvinylidene fluoride membrane. The membrane was blocked with polyvinyl alcohol and then probed overnight at 4°C with: rabbit polyclonal antibodies against AMPK, cleaved CASPASE-3 (1∶1000 dilution, Cell Signaling Technology, Danvers, MA), AGC1 (1∶7500, provided by J. Satrustegui, University of Madrid), AGC2 (1∶5000, [32]), GC1 (1∶5000, [21]), CAC, CIC and DIC (1∶10000, provided by F. Palmieri, University of Bari); mouse monoclonal antibodies against SIRT1 (1∶1000), ACTIN (1∶200000, Chemicon-Millipore, Zug, Switzerland), TUBULIN (1∶100000, Sigma-Aldrich) and 5 subunits of OXPHOS complexes (1∶15000, MitoSciences, Eugene, OR); goat polyclonal antibodies against SIRT4 (1∶1000, Abcam Inc., Cambridge, MA), PGC1α, TFAM, UCP2 (1∶1000, Santa Cruz Biotechnology, Santa Cruz, CA) and PDX1 (1∶50000, provided by C. Wright, Vanderbilt University). After washing, the membrane was incubated 1 h at RT with secondary horseradish peroxidase-conjugated anti-rabbit, anti-mouse or anti-goat antibodies IgG (1∶10000, Amersham Biosciences, UK) according to primary antibodies. Proteins were visualized by chemiluminescence (ECL #RPN2135, Amersham), analyzed with the ChemiDoc XRS System (Bio-Rad, Hercules, CA) and bands were quantified with Scion Image software (Scion Corporation, Frederick, MD).

### Cytoscape

Gene and protein data obtained from Mito-array and immunoblotting were analyzed by integrating knowledgebase (UniProtKB/Swiss-Prot/neXtProt) data [33] with differential sub-cellular expression information to establish associations between genes and proteins. Visualization of relationships between genes was drawn with the Cytoscape software version 2.8.2 [34].

### Statistical analysis

All data were analyzed with the IBM SPSS Statistics 19.0 software (SPSS, Chicago, IL). Statistical tests between each stress condition and the control G11 values were performed using one-way ANOVA analysis followed by Dunnet post hoc test. Results are presented as mean ± SEM. A *P* value lower than 0.05 was considered statistically significant.

## Results

### Effects of metabolic stresses on cell function

In order to compare the chronic effects of different metabolic stresses in parallel and their respective contribution to β-cell dysfunction, expression of key components of GSIS was determined in INS-1E cells. Because INS-1E cells are normally cultured at 11.1 mM glucose, the effects of metabolic stresses were compared to this standard culture condition (G11), considered as safe glucose level for these cells. Insulin secretion from G11 cells (Figure S1) evoked by 15 mM glucose was stimulated 3.0-fold *versus* basal release. Culturing cells at low (G5.6) and high (G25) glucose concentrations during 3 days did not modify basal release but reduced GSIS by 53% and 57% *versus* corresponding G11 control, respectively (1.8-fold response for both conditions). As expected [30], cells exposed to palmitate (Palm) and oleate (Olea) increased basal insulin release (+120% and +139%, respectively). Palmitate and oleate treated cells responded to 15 mM glucose, with 2.1-fold (Palm) and 1.7-fold (Olea) increases in insulin release. GSIS was markedly reduced in INS-1E cells after oxidative stress (H_2_O_2_) compared to G11 control [16].

**Figure 1:**
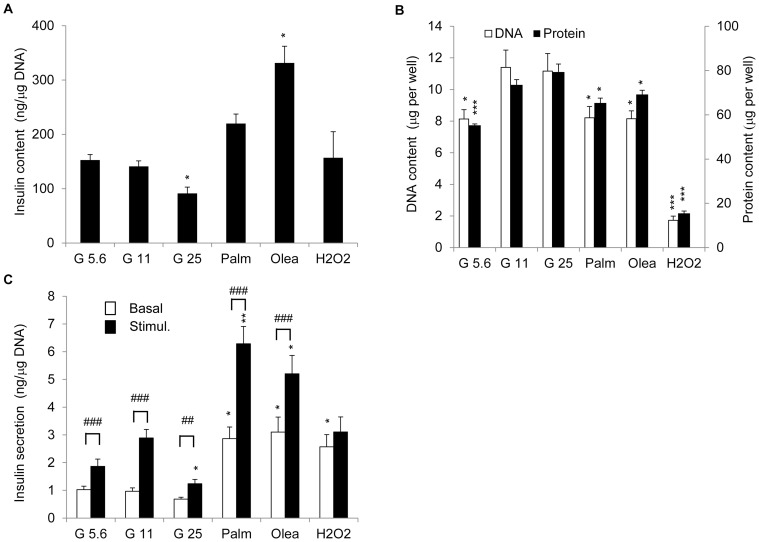
Secretory responses and insulin/DNA/protein contents of INS-1E cells after stress exposure. INS-1E cells were exposed for 3 days to different culture conditions: 5.6 mM glucose (G5.6), 25 mM glucose (G25), 0.4 mM palmitate (Palm), 0.4 mM oleate (Olea). Culture at 11.1 mM glucose (G11) served as no stress (negative control) and transient oxidative stress at day 0 (200 µM H_2_O_2_ for 10 min) served as acute stress (positive control). At day 3, cells were washed and insulin secretion was measured at basal 2.5 mM (Basal, white bars) and stimulatory 15 mM glucose concentrations (Stimul., black bars) following a 30 min incubation period. (A) Insulin and (B) DNA (white bars), protein (black bars) contents at the end of the culture period. (C) Secretory responses were normalized to DNA content. Values are means ± SEM of 6 independent experiments, each done in duplicate. *P<0.05, **P<0.01, ***P<0.005 *versus* corresponding G11 controls; # *P*<0.05, ## *P*<0.01, ### *P*<0.005 *versus* corresponding basal secretions.

Insulin content was decreased by 35% in G25 cells and was increased by 135% in cells exposed to oleate (Olea), compared to G11 controls (Figure 1A). INS-1E cells exposed to different stresses for 3 days exhibited lower DNA and protein contents, reflecting a reduction in cell number (Figure 1B), except for G25 cells. When insulin secretion was normalized to DNA contents, in other words by cell number, we observed similar inhibition of GSIS *versus* G11 control cells: −39% in G5.6, −40% in G25, −27% in Palm, −43% in Olea and −60% in H_2_O_2_ (Figure 1C). In the case of cells exposed to fatty acids and oxidative stress, these reduced fold responses were mainly explained by elevated basal insulin release (+196% in Palm, +220% in Olea, +165% in H_2_O_2_ *versus* G11 control basal release; Figure 1C). Incidentally, these data confirmed that 11.1 mM is the optimal glucose concentration for INS-1E culture in terms of cell number (Figure 1B) and GSIS (Figure 1C).

### Effects of metabolic stresses on cell viability and stress response pathways

**Figure 2:**
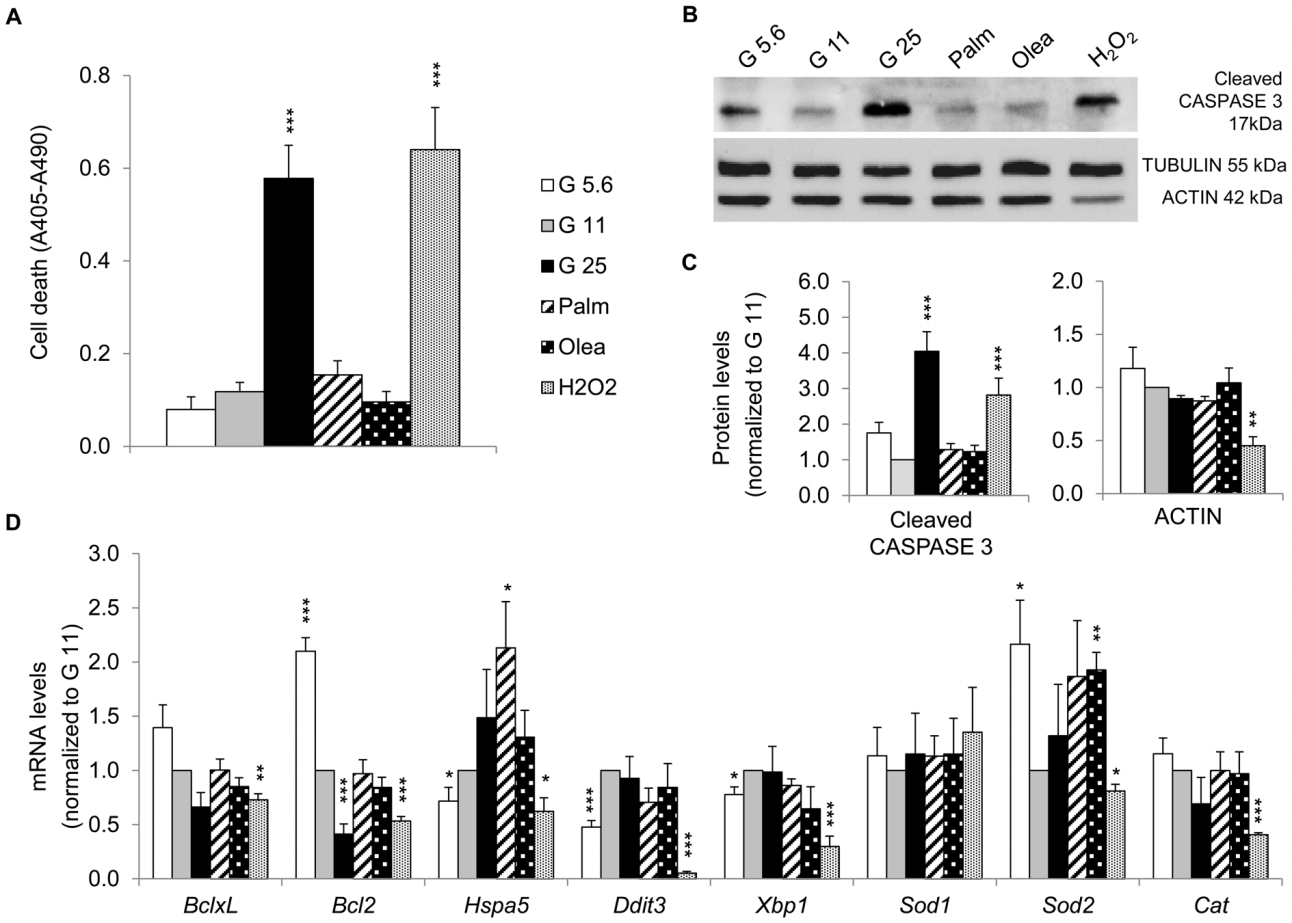
Apoptosis and expression profile of genes involved in stress response pathways after stress exposure. (A) Cell death of INS-1E cells cultured without stress at 11.1 mM glucose concentration (G11, control) or exposed to different culture conditions listed in legend of figure 1. Data are shown as means ± SEM of 4 independent experiments. (B) Representative immunoblotting showing levels of the cleaved CASPASE 3, TUBULIN and ACTIN from treated INS-1E cells. (C) Quantitative analysis of band densities normalized to TUBULIN from immunoblots as shown in (B) is presented as means ± SEM of 4 independent experiments. Results are expressed as protein levels normalized to the control value of G11. (D) Transcript levels normalized to those of 18S and expressed as changes *versus* value of G11. The *BclxL* and *Bcl2* genes encode antiapoptotic proteins; the *Hspa5*, *Ddit3* and *Xbp1* genes encode the endoplasmic reticulum stress related proteins BIP, CHOP and XBP1, respectively; the cytosolic isoform *Sod1*, the matrix isoform *Sod2* (superoxide dismutase) and *Cat* (catalase) genes encode antioxidative enzymes. Results are means ± SEM of 2 independent experiments done in triplicate. *P<0.05, **P<0.01, ***P<0.005 *versus* G11 controls.

To investigate whether the observed β-cell dysfunction was associated with cytotoxic effects in our culture conditions, cell death was quantified at the end of the 3 days of stress exposure. Control G11 cells exhibited basal apoptotic rate (Figure 2A), in the range of 3–4% [30]. As opposed to G5.6, G25 induced marked increase in cell death (4.9-fold *versus* G11), similar to oxidative stress effects (5.4-fold *versus* G11) [16]. Lipotoxicity associated with apoptosis is regularly observed with saturated fatty acids [8]–[10], whereas unsaturated fatty acids promote mostly impairment of GSIS [5]–[7]. In cultured cells and isolated islets, fatty acid treatments triggering apoptosis have usually been performed in the presence of low FCS (1%) or even the absence of serum. When cultured with standard higher FCS concentrations (5–10%), longer incubation periods are required to observe cytotoxic effects [11], [12]. We chose to perform all treatments at 5% FCS in order to investigate the intrinsic effects of saturated *versus* unsaturated fatty acids without changing the standard culture conditions. In accordance with previous reports [7], [30], oleate did not increase cell death. In our hands, palmitate did not induce significant cytotoxicity, as confirmed by assessment of CASPASE 3 cleavage (Figure 2B, C). High glucose and oxidative stress promoted apoptosis (4.1-fold cleaved CASPASE 3 in G25 and 2.8-fold in H_2_O_2_ *versus* G11 control). Interestingly, oxidative stress induced a decrease in ACTIN levels, commonly used as loading control, whereas TUBULIN levels were not affected. We also examined the expression profile of genes involved in stress response pathways, such as unfolded protein response (UPR) and antioxidant defenses. Treatment of cells with G5.6 for 3 days enhanced anti-apoptotic *Bcl2* expression (Figure 2D), along with decreased expression of UPR genes (*Hspa5*/BIP, *Ddit3*/CHOP, *Xbp1*) and increased mitochondrial superoxide dismutase *Sod2* mRNA. G25 lowered *Bcl2* expression, without changing UPR and antioxidant markers. Palmitate and oleate increased the mRNA levels of the ER chaperon protein *Hspa5*/BIP and the antioxidant enzyme *Sod2*, suggesting specific protective responses according to the nature of fatty acids. There was no change in mRNA levels of *Ddit3*/CHOP and *Xbp1* (Figure 2D), two transcription factors mediating ER stress-induced apoptosis [35], [36], consistent with preserved cell viability. Oxidative stress caused a drastic reduction in the expression profile of genes involved in stress responses (Figure 2D). Overall, high glucose promoted cell death and mainly affected anti-apoptotic pathway through down-regulation of *Bcl2*, recapitulating characteristics of glucotoxicity; whereas fatty acids induced lipo-dysfunction.

### Expression of energy sensors and transcription factors

**Figure 3:**
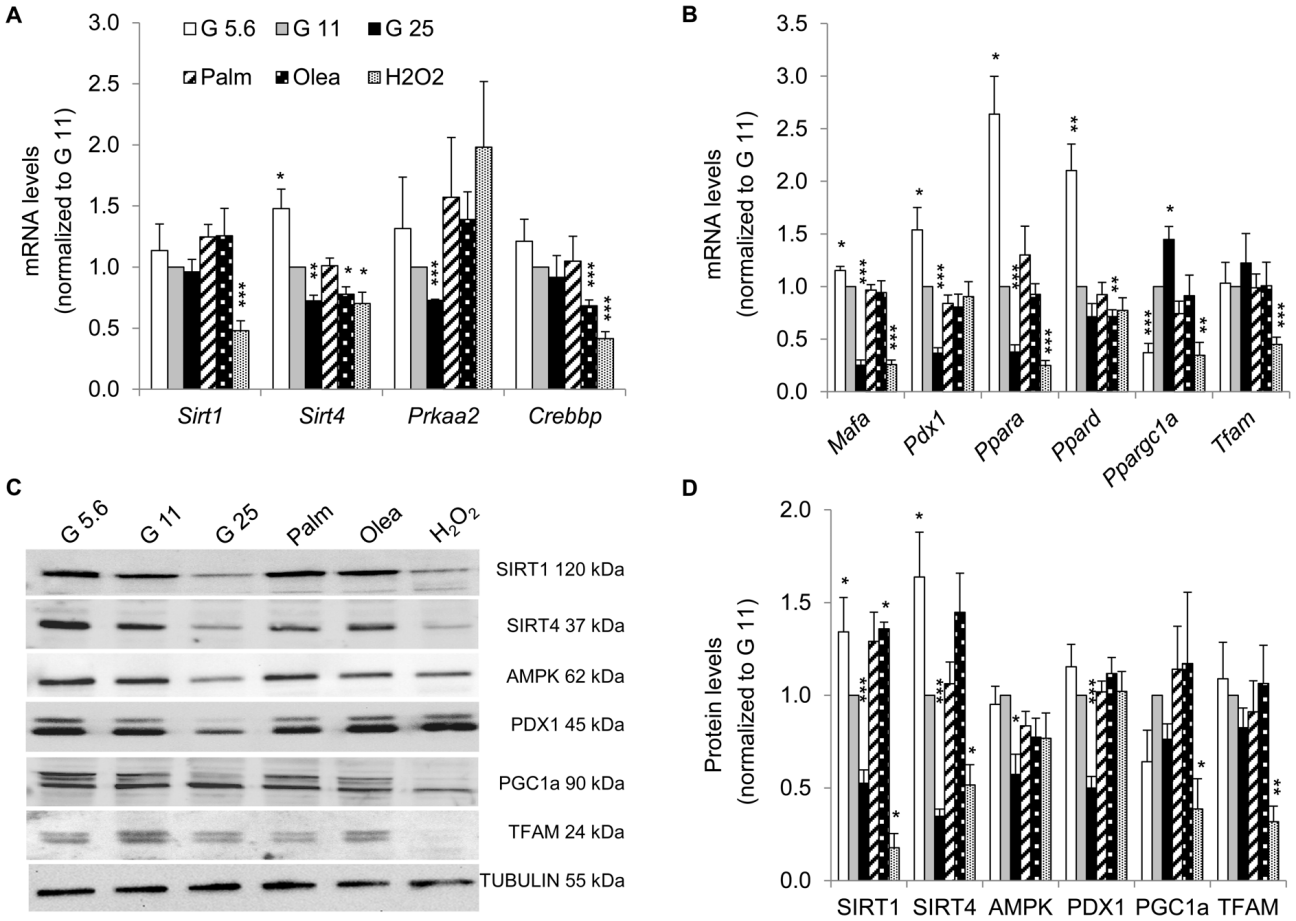
Expression of key energy sensors and transcription factors in INS-1E cells after stress exposure. Transcript levels of (A) redox state-related genes (the sirtuins *Sirt1* and *Sirt4*), the catalytic subunit of AMPK encoded by *Prkaa2* gene, the cAMP-responsive acetylase CREB binding protein *Crebbp* and (B) transcription factors were quantified as described for figure 2D. Results are means ± SEM of 2 independent experiments done in triplicate. (C) Representative immunoblotting showing levels of the energy sensors SIRT1, SIRT4, AMPK, PGC1α and the transcription factors PDX1 and TFAM from INS-1E cells under different experimental conditions. (D) Quantitative analysis of relative band densities normalized to TUBULIN as shown in (C) is presented as means ± SEM of at least 4 independent experiments. Results are expressed as protein levels normalized to G11 values. *P<0.05, **P<0.01, ***P<0.005 *versus* G11 controls.

Expression of essential β-cell genes were repressed by high glucose (G25); such as the NAD-dependent sirtuin *Sirt4* and the catalytic subunit of AMP-activated protein kinase *Prkaa2* (Figure 3A), the transcription factors *Mafa* and *Pdx1* involved in insulin expression, and the nuclear receptor *Pparα* regulating fat metabolism (Figure 3B). Interestingly, the mirror image was induced by G5.6, indicating that glucose dose-dependently regulated this set of genes in INS-1E cells. We extended this analysis at the protein level and found that SIRT1, SIRT4, AMPK, and PDX1 were decreased in glucotoxic conditions (Figure 3C, D). In conditions inducing lipo-dysfunction, only oleate treatment affected energy sensing by downregulation of *Sirt4*, *Crebbp* and *Ppar*δ and upregulation of SIRT1. In agreement with previous report [16], oxidative stress decreased expression of *Tfam* and *Ppargc1α* (PGC1α), two factors responsible for mitochondrial biogenesis, providing validation of our Mito-array data (Figure 3B). This observation was substantiated at the protein level (Figure 3C, D) showing lower levels of PGC1α and TFAM. Additionally, immunoblotting revealed novel targets of oxidative stress including SIRT1 and SIRT4. Expressions of AMPK and PDX1 were not altered by H_2_O_2_ treatment, indicating selective effects induced by oxidative stress. Taken together, data show that glucose regulated key transcription factors and energy sensors, both at the mRNA and protein levels, while fatty acids affected very modestly the transcriptional machinery and the energy sensing capacity.

### Expression profile of genes implicated in glucose metabolism

**Figure 4:**
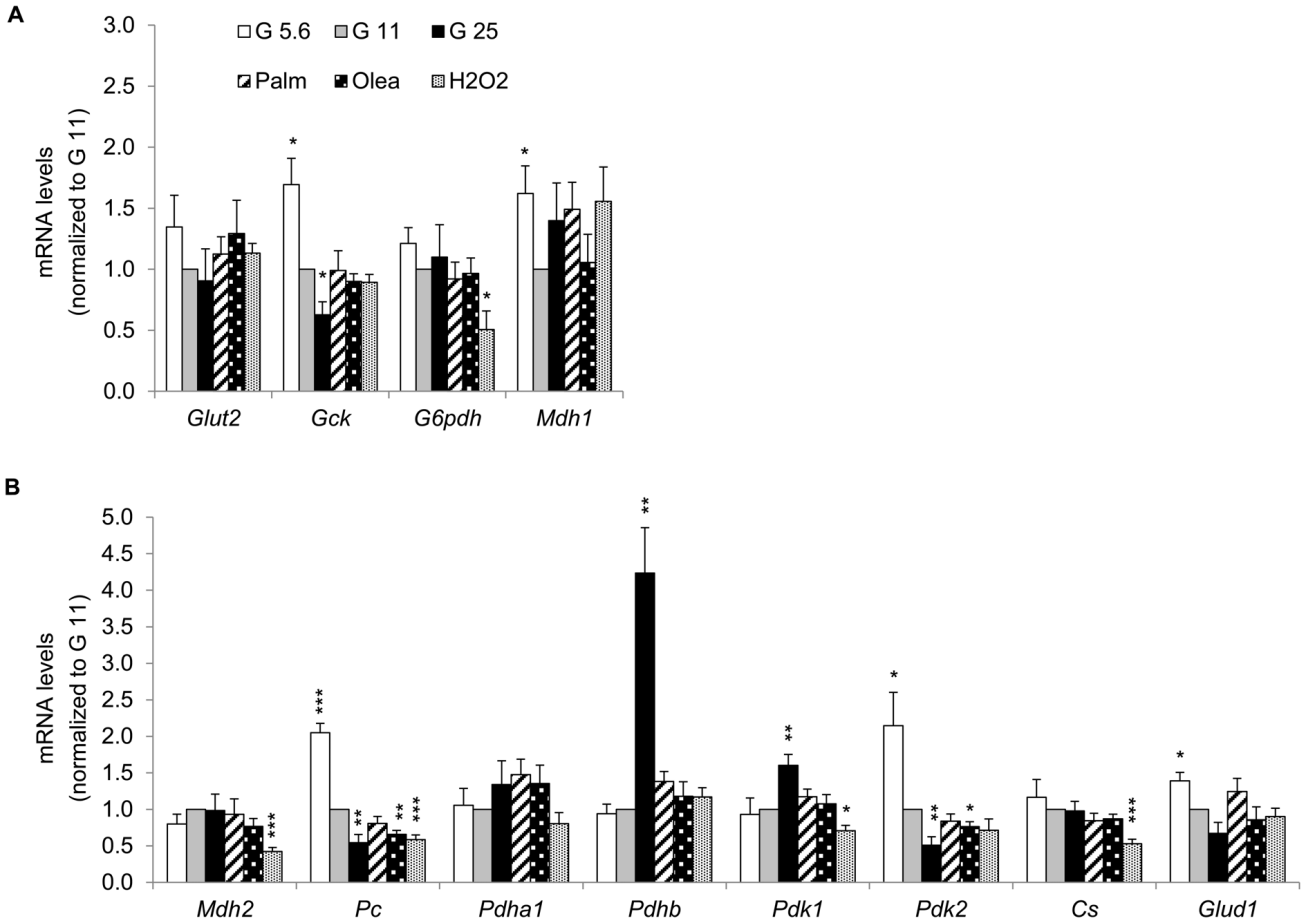
Expression profile of genes implicated in glucose metabolism in INS-1E cells after stress exposure. Transcript levels of genes encoding (A) cytosolic and (B) mitochondrial proteins were quantified as described in figure 2D. (A) The genes *Glut2* (glucose transporter 2), *Gck* (glucokinase), *G6pdh* (glucose-6-phosphate dehydrogenase) and *Mdh1* (malate dehydrogenase 1) encode cytosolic proteins; (B) the genes *Mdh2*, *Pc* (pyruvate carboxylase), *Pdha1*, *Pdhb* (pyruvate dehydrogenase subunit alpha and beta, respectively), *Pdk1*, *Pdk2* (pyruvate dehydrogenase kinase 1 and 2), *Cs* (citrate synthase) and *Glud1* (glutamate dehydrogenase 1) encode mitochondrial enzymes. Results are means ± SEM of 2 independent experiments done in triplicate. *P<0.05, **P<0.01, ***P<0.005 *versus* G11 controls.

Next, we analyzed the expression of key genes for β-cells encoding cytosolic and mitochondrial proteins involved in glucose sensing. Compared to G11, G5.6 and G25 induced an inverse glucose dose response expression of glucokinase (*Gck*), pyruvate carboxylase (*Pc*), pyruvate dehydrogenase kinase (*Pdk2*) and glutamate dehydrogenase (*Glud1*) (Figure 4A, B). Consistent with moderate effects induced by fatty acids on the transcription factors, only oleate (not palmitate) slightly decreased mRNA levels of *Pc* and *Pdk2* (Figure 4B). In a previous study, *Pc* was shown to be down-regulated by both palmitate and oleate in the Min6 β-cell line [37]. Oxidative stress caused a selective repression of genes encoding for cytosolic glucose-6-phosphate dehydrogenase *G6pdh* and for mitochondrial malate dehydrogenase *Mdh2*, citrate synthase *Cs*, *Pc, Pdk1* (Figure 4A, B). Our results show that chronic exposure to high glucose caused a selective downregulation of cytosolic and mitochondrial enzymes involved in glucose metabolism.

### Expression of mitochondrial respiratory chain subunits and associated carriers

**Figure 5:**
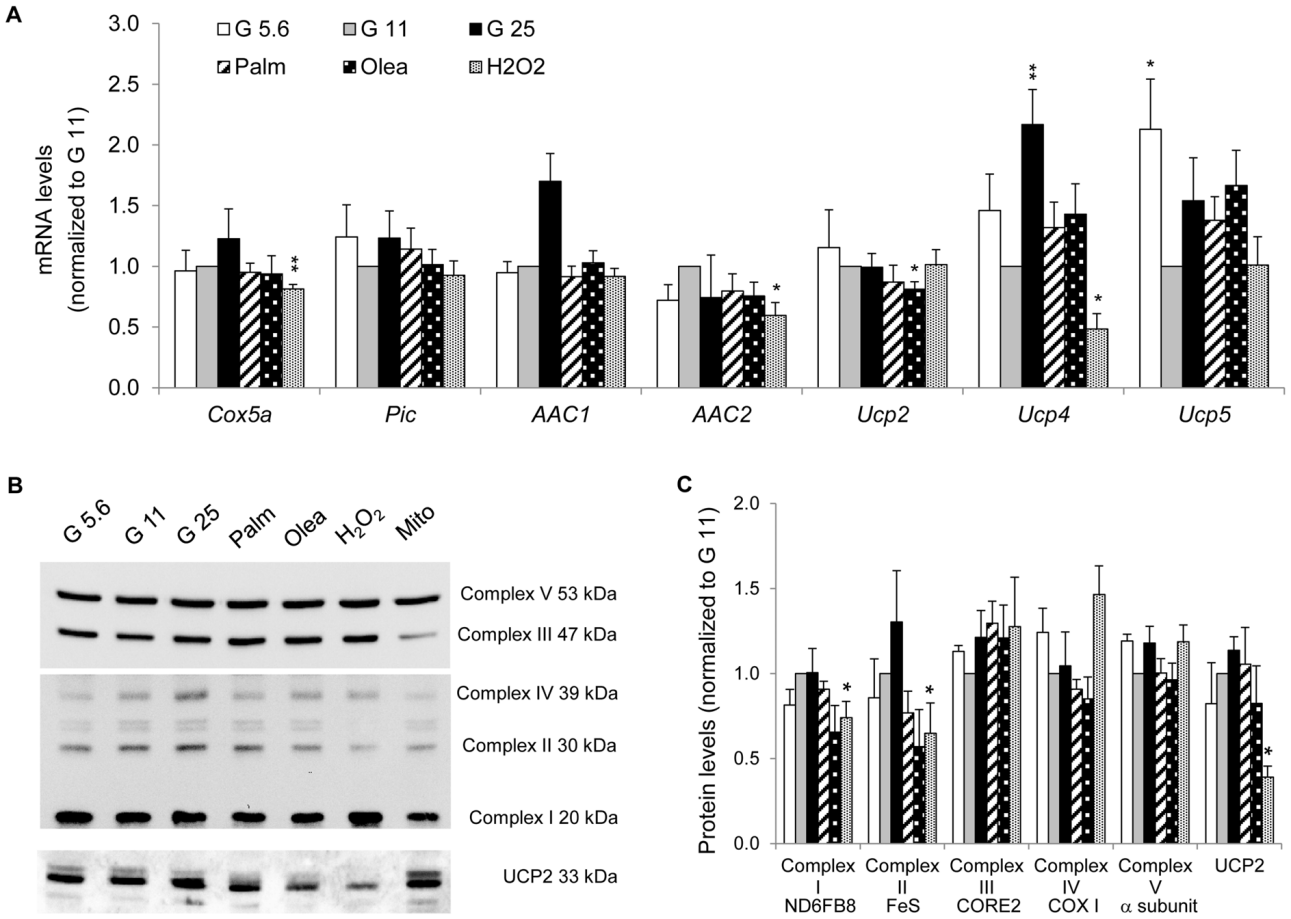
Expression of mitochondrial respiratory chain subunits and OXPHOS associated carriers in INS-1E cells after stress exposure. (A) Transcript levels of *Cox5a* (complex IV), the phosphate carrier *Pic*, the ADP/ATP carriers *AAC1* and *AAC2*, the uncoupling proteins *Ucp2*, *Ucp4* and *Ucp5* were quantified as described in figure 2D. Results are means ± SEM of 2 independent experiments done in triplicate. (B) Immunoblotting showing protein subunits of the five OXPHOS complexes and UCP2 (total cell extracts). Additionally, mitochondria (Mito) isolated from G11 cells were used as control. (C) Quantitative analysis of relative band densities normalized to TUBULIN is presented as means ± SEM of at least 3 independent experiments. Results are shown as protein levels normalized to G11 values. *P<0.05, **P<0.01 *versus* G11 controls.

**Figure 6:**
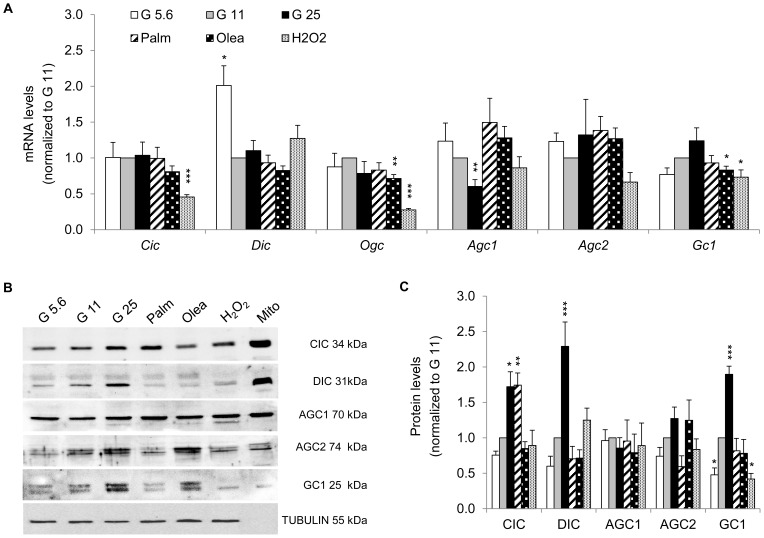
Expression of mitochondrial metabolite carriers implicated in redox pathways in INS-1E cells after stress exposure. (A) Transcript levels of the carriers for citrate/isocitrate *Cic*, dicarboxylate *Dic*, 2-oxoglutarate/malate *Ogc*, aspartate/glutamate *Agc1* and *Agc2*, glutamate *Gc1* were quantified as detailed in figure 2D. Results are means ± SEM of 2 independent experiments done in triplicate. (B) Representative immunoblotting showing protein levels of the metabolite carriers CIC, DIC, AGC1, AGC2 and GC1 from INS-1E cells (total extracts). Mitochondrial origin was confirmed on mitochondria (Mito) isolated from control G11 cells. (C) Quantitative analysis of band densities normalized to TUBULIN from immunoblots as shown in (B) is presented as means ± SEM of at least 3 independent experiments. Results are expressed as protein levels normalized to G11 values. *P<0.05, **P<0.01, ***P<0.005 *versus* G11 controls.

**Figure 7:**
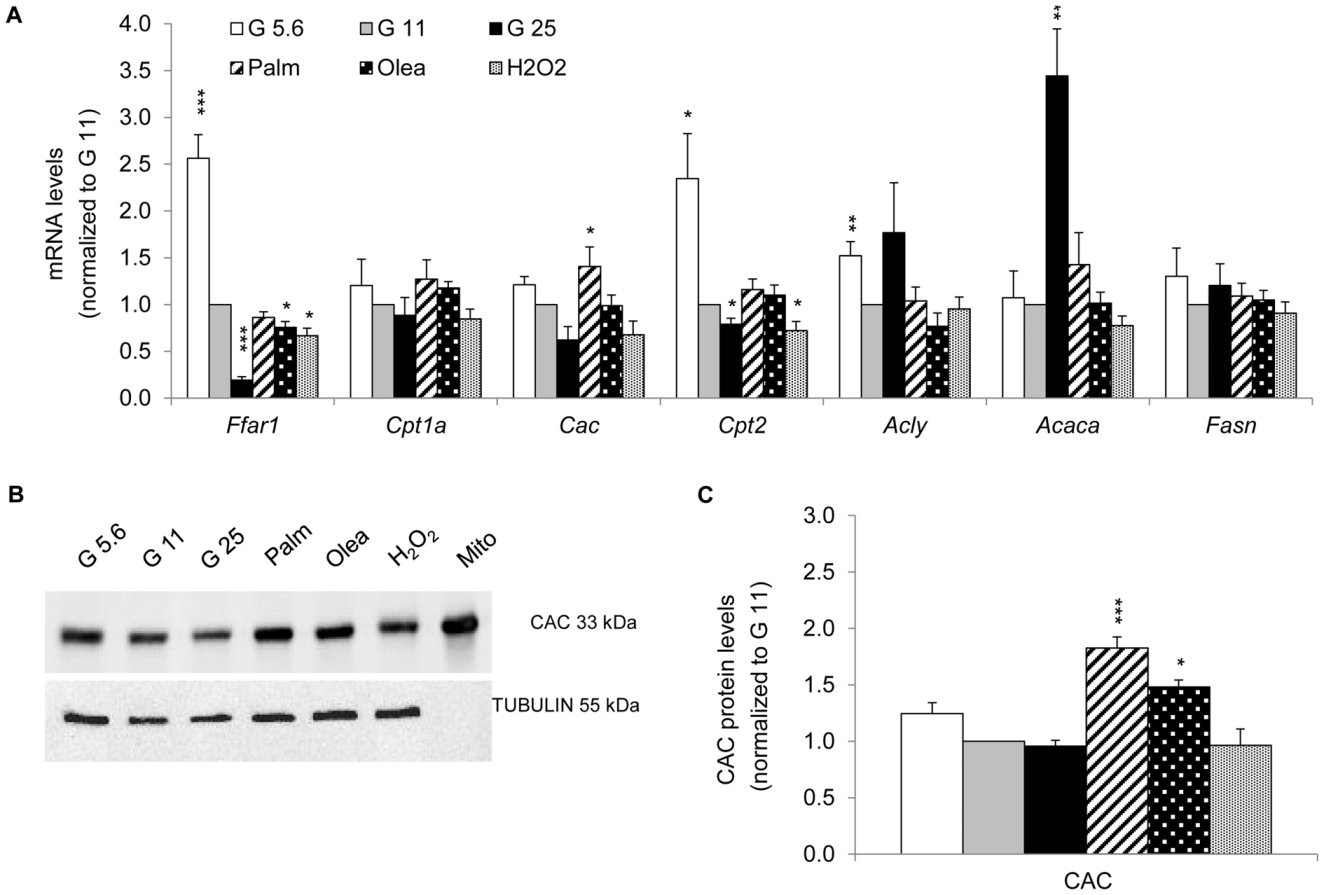
Expression of the carnitine/acylcarnitine carrier *Cac* and genes involved in lipid metabolism after stress exposure. (A) Gene transcripts implicated in lipid pathways (free fatty acid receptor 1 GPR40 *Ffar1*, carnitine O-palmitoyltransferase 1 *Cpt1a* and 2 *Cpt2*, carnitine/acylcarnitine carrier *Cac*) and in *de novo* lipid synthesis (ATP-citrate lyase *Acly*, acetyl-CoA carboxylase *Acaca*, fatty acid synthase *Fasn*) were quantified as described in figure 2D. Results are means ± SEM of 2 independent experiments done in triplicate. (B) Immunoblotting showing levels of CAC protein from INS-1E cells (total extracts). Mitochondrial origin of CAC was confirmed on mitochondria (Mito) isolated from control G11 cells. (C) Quantitative analysis of band densities normalized to TUBULIN from immunoblots as shown in (B) is presented as means ± SEM of 4 independent experiments. Results are expressed as protein levels normalized to G11 value. *P<0.05, **P<0.01, ***P<0.005 *versus* G11 controls.

In order to document the expression profile of the mitochondrial machinery, both in healthy and stressed cells, we analyzed transcript and protein levels of carriers of the *Slc25* gene family in INS-1E cells. Among the 27 *Slc25* genes analyzed by the Mito-array, we identified 22 mitochondrial carriers being expressed at mRNA level (Figures 5 to 7 and S2). Genes with Ct values equal or higher than 35 were considered below physiological levels and were not included in the final analysis. These include the uncoupling proteins UCP1 (*Slc25a7*) and UCP3 (*Slc25a9*), the glutamate carrier GC2 (*Slc25a18*), the oxodicarboxylate carrier (*Slc25A21*) and the solute carrier *Slc25a34*. Mitochondrial carriers can be grouped according to their cellular functions [18], [19]. Several carriers are associated with ATP production or uncoupling function. None of the metabolic stresses changed expression of mitochondrial respiratory chain subunit IV *Cox5a* or OXPHOS associated carriers, *i*.*e*. inorganic phosphate carrier *Pic* and ADP/ATP carriers *AAC1* (*Slc25a4*) and *AAC2* (*Slc25a5*) (Figure 5A). Immunoblotting confirmed preservation at the protein level of the respiratory chain complex subunits (Figure 5B, C). By contrast, oxidative stress reduced *Cox5a* mRNA levels as well as ND6FB8 (complex I) and FeS (complex II) protein levels. Interestingly, we observed a specific decrease in UCP2 (*Slc25a8*) protein levels secondary to oxidative stress.

### Expression of mitochondrial solute carriers responsible for metabolite transport

The second group of *Slc25* mitochondrial carrier family mediates solute transport across the inner mitochondrial membrane. The regulation of expression of this family is still poorly characterized, in particular under metabolic stresses. We first assessed the expression of solute carriers known to be involved in GSIS [21]–[26], [32], [38], both at the mRNA (Figure 6A) and protein levels (Figure 6B, C). G25 up-regulated protein levels of the citrate/isocitrate carrier CIC, the dicarboxylate carrier DIC, and the glutamate carrier GC1 (Figure 6B, C), whereas corresponding mRNA levels were unchanged (Figure 6A). Lower GC1 observed at G5.6 indicates that glucose dose-dependently regulated glutamate transport. At the protein level, solute carriers remained unchanged in INS-1E cells exposed to fatty acids, with the exception of CIC being up-regulated after palmitate treatment. Oxidative stress caused a marked decrease of GC1.

### Expression of the mitochondrial carriers and genes involved in lipid metabolism

Finally, we studied the expression of genes implicated in lipid pathways. G25 induced a 3.2-fold increase in the lipogenic enzyme acetyl-CoA carboxylase *Acaca*/ACC mRNA [39], whereas transcript levels of the free fatty acid receptor 1 *Ffar1*/GPR40 and the mitochondrial carnitine O-palmitoyltransferase 2 *Cpt2* were reduced (Figure 7A). Interestingly, the inverse transcriptional changes were observed at G5.6, showing glucose dose-dependent regulation of *Ffar1* and *Cpt2*. Exposure to fatty acids did not change transcripts of lipogenic genes (Figure 7A). We measured both mRNA and protein levels of carnitine/acylcarnitine carrier CAC, the rate-limiting mitochondrial transporter of fatty acids for β-cell oxidation. There was a trend for glucose dose-dependent decrease in CAC expression, whereas palmitate significantly increased mRNA levels of *Cac*. At the protein level, CAC was induced by palmitate and oleate (Figure 7B, C). The results show that fatty acids up-regulated CAC expression, whereas glucose slightly repressed CAC.

## Discussion

The respective contribution of the different diabetes-associated stresses to dysfunction and death of insulin-secreting cells remains unclear. In particular, can we identify stress-specific signatures for high glucose concentrations (glucotoxicity), saturated or unsaturated fatty acids (lipotoxicity and/or lipo-dysfunction) and oxidative stress? Here, we explored molecular targets potentially specific to, or shared by, different metabolic stresses investigated side-by-side in highly standardized conditions on INS-1E cells.

**Figure 8:**
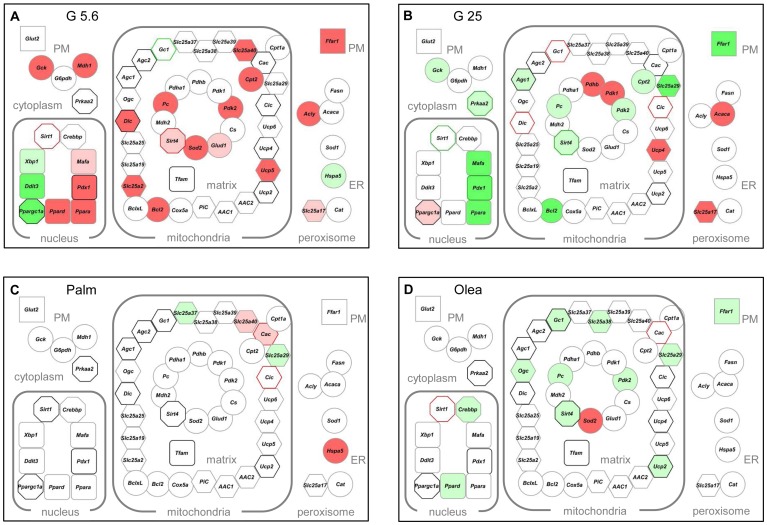
Transcriptome and proteome from INS-1E cells cultured 3 days under different stress conditions. The schemes provide a global view of the expression of the 60 genes at transcript (node core) and protein (node border) levels: (A) low glucose (G5.6) (B) high glucose (G25) (C) palmitate (Palm) (D) oleate (Olea). The expressed genes were grouped using the Cytoscape software according to their protein subcellular localization (from the databases UniProtKB/SwissProt and neXtProt); plasma membrane (PM), cytoplasm, nucleus, mitochondrial inner membrane, matrix, endoplasmic reticulum (ER), and peroxisome. Node shape: rectangles represent transporters or receptors, circles are enzymes or stress proteins, octagons show energy related sensors, round rectangles transcription factors, and hexagons carriers. Colors reflect changes in expression levels *versus* G11 controls: green and red for significant (*P*<0.05) down- and upregulation, respectively. Dark green: levels <0.5; light green: levels >0.5 but <0.8; pink: levels >1.2 but <1.5; red: levels >1.5. Border colors: black no change in protein level; grey not tested.

Characterization of mitochondrial carriers over the last decades demonstrated their implication in numerous metabolic pathways and their alteration in several diseases. The role of some mitochondrial carriers in metabolism-secretion coupling has been substantiated only recently (reviewed in [40]). Figure 8 shows the identified 22 mitochondrial carriers being expressed in INS-1E cells, as well as their transcriptional regulation after stress exposure (Figure S3).

### Mitochondrial markers of low glucose

**Figure 9:**
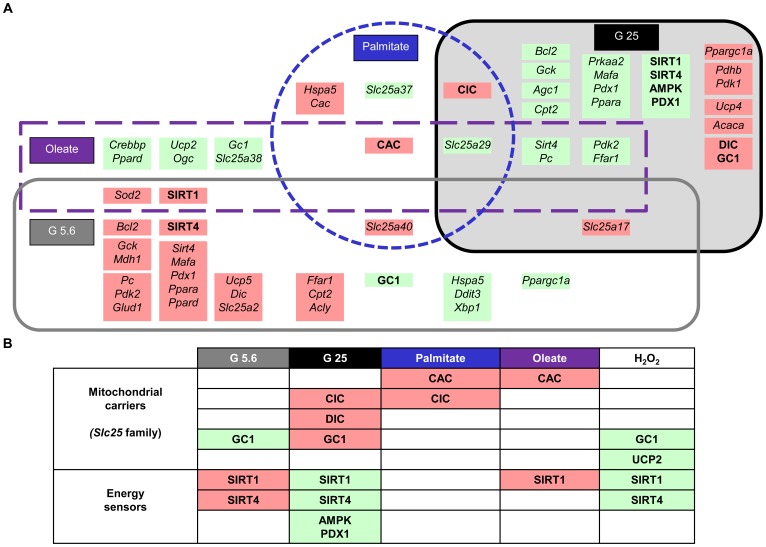
Overview of alterations in INS-1E cells induced by the different metabolic stresses, delineating stress-specific signatures. Green and red colors refer to significant (*P*<0.05) down- and upregulation, respectively, of mRNA or proteins levels *versus* G11 controls. Capital bold letters represent proteins and small case italic names represent transcripts.

Both hypo- and hyperglycemia alter the coupling of glucose metabolism to insulin secretion, induce the loss of β-cell differentiation, and increase the rate of cell death [4]. Similarly, *in vitro* exposure to either low or high glucose markedly impairs rat β-cell function and survival [41]. Figures 8A and 9A summarize the transcriptome and proteome in G5.6, i.e. low glucose levels for INS-1E cells although above hypoglycemia threshold. The glucose-dependent regulation of insulin expression is primarily mediated by the transcription factors PDX1 and MAFA, both being induced by G5.6 at transcript level. PDX1 and MAFA are also master regulators of genes implicated in β-cell function; such as the glucose transporter *Glut2*, the glucose sensor glucokinase and the pyruvate carboxylase [42], [43]. Consistently, the two latter genes were up-regulated in G5.6 (Figure 8A). A striking observation was the increased expression of the sirtuins SIRT1 and SIRT4 in G5.6. As sirtuin activity depends on NAD^+^/NADH ratio, they are sensitive to the cellular redox state and serve as energy sensors [44], [45]. SIRT1 is mainly found in the nucleus where it acts as a transcriptional activator *via* deacetylation activity. In the β-cell, the main SIRT1 targets are HNF-1α and PDX1. SIRT4 is located in the mitochondrial matrix and regulates glutamate dehydrogenase 1 (*Glud1*/GDH) activity [46]. Here, we observed glucose-dependent regulation of SIRT1 and SIRT4, suggesting a sequence of events in which chronic low glucose increases SIRT1 level, inducing *Pdx1* and possibly *Mafa*. This in turn might promote expression of *Glut2*, *Gck*, *Pc*, and insulin. Therefore, INS-1E cells adapted to hypoglycemic condition, partially preserving their phenotype. Another novel finding was the observed glucose-dependent regulation of mitochondrial carriers CIC, DIC, AGC2 and GC1 at the protein level. At G5.6, only GC1 was down-regulated, conferring to this glutamate carrier a specific signature and target of hypoglycemic conditions. In β-cells, glutamate acts downstream of mitochondrial function, participating in the coupling of glucose metabolism to insulin secretion [19], [47]. Glutamate metabolism is tightly controlled by activities of mitochondrial enzymes and carriers (reviewed in [48]), in particular GDH and GC1, while AGC1 is mainly involved in transamination reactions of the malate-aspartate shuttle [40]. GC1 is present in insulin-secreting cells and inhibition of its activity decreases glutamate transport and GSIS [21]. In G5.6, GC1 protein levels were markedly reduced compared to control cells. One can speculate that down-regulation of GC1 in hypoglycemic conditions could reduce glutamate transport and GSIS similarly to GC1 silencing. Overall, G5.6 induced down-regulation of 4 genes and up-regulation of 20 genes at mRNA level, protein levels of SIRT1 and SIRT4 being increased and of GC1 being decreased, providing a stress-specific signature to hypoglycemic conditions (Figure 9B).

### Mitochondrial markers of glucotoxicity

In line with previous results [49]–[51], chronic exposure of INS-1E cells to G25 decreased GSIS and insulin content, altered differentiation *via* decreased expression of PDX1 and *Mafa*, and induced CASPASE 3 cleavage and cell death. Chronic high glucose modifies transcript levels of metabolic enzymes and transcription factors [39], [51], [52]. Recent proteomic analyzes provided new insights into changes induced by glucotoxicity [50], [53], although mitochondrial alterations are still poorly characterized. Interestingly, inverse transcriptional changes were observed in G25 *versus* G5.6, indicating that glucose dose-dependently regulated a specific set of genes in INS-1E cells (Figures 8B and 9A). Chronic exposure to high glucose may saturate the TCA cycle and respiratory chain, favoring cataplerotic generation of mitochondrial metabolites, such as citrate and glutamate, along with elevation of NADH and NADPH through shuttle activities [54], [55]. Malate transport by DIC is a critical shuttle component for NADPH production mediated by pyruvate cycling, participating to GSIS [25]. CIC-mediated pyruvate–isocitrate cycling also plays an important role in GSIS [23], [56]. CIC is essential for cytosolic malonyl-CoA and fatty acid synthesis and also for cytosolic NADPH production. Because CIC requires cytosolic malate as a counter-substrate for citrate and isocitrate export out of mitochondria, expression of CIC and DIC could be similarly regulated by glucose. Accordingly, we observed parallel up-regulation of CIC, DIC and GC1 by G25. G25 also modified the redox-dependent sirtuins, both nuclear SIRT1 and mitochondrial SIRT4, as well as the AMPK protein. Figure 9B summarizes the stress-specific signature for hyperglycemic conditions.

### Mitochondrial markers of saturated and unsaturated fatty acids

It is generally accepted that saturated fatty acids, such as palmitate, promote lipotoxicity that may result into apoptosis, whereas unsaturated fatty acids, such as oleate, induce β-cell dysfunction characterized by elevated basal insulin release and impaired GSIS [8], [30], [57]. Here, we observed that palmitate and oleate treatments in the presence of standard FCS concentration increased the basal more than the stimulated release of insulin, resulting in blunted glucose response. This is consistent with a recent study in INS-1 832/13 cells exposed to palmitate for 48h, showing preserved cell viability and insulin content along with impaired GSIS [11]. Incubation with palmitate in the absence or reduced concentrations of FCS (1%) promotes cell death [8]–[10]. Here, with standard 5% FCS no significant cytotoxic effects were observed. Thus, the lipo-dysfunction developed by INS-1E cells is similar to the ones reported for primary islets and mouse insulinoma [58], [59], as well as for islets from patients with type 2 diabetes [60]. Palmitate (Figure 8C) and oleate (Figure 8D) modestly modified the transcriptional machinery, the energy sensing capacity, and the mitochondrial carriers. However, we identified specific targets according to the nature of the fatty acids (Figure 9A, B). Indeed, the protein levels of CIC and SIRT1 were specifically up-regulated by respectively palmitate and oleate. SIRT3 has been reported to reduce palmitate-induced oxidative stress in kidney cells [61]. In skeletal muscles of diabetic mice, SIRT3 expression is reduced, increasing oxidative stress and altering insulin signaling [62]. Recently, islets and parental INS1 cells were shown to express SIRT3 that was down-regulated following exposure to cytokines, indicating a protective role in inflammatory conditions [63].

Both fatty acids increased expression of CAC, an effect also observed in Min6 cells [37]. CAC catalyzes transport of acylcarnitine into mitochondria and is part of the carnitine shuttle system, also comprising CPT1 and CPT2, and is rate-limiting for β-oxidation [19]. Fibrates induce *Cac* expression by binding to PPARα, a transcription factor known as a lipid sensor [64]. Up-regulation of PPARα protects INS-1E cells from oleate-induced dysfunction, preserving the glucose response and promoting fatty acid turnover [30]. The present data indicate that CAC can be up-regulated without necessarily requiring increased expression of PPARα, for instance by fatty acid-induced activation of existing PPARα.

## Conclusion

In the context of diabetes in general and β-cell dysfunction in particular, various conditions have been proposed to trigger damages of insulin-secreting cells. Among them, chronic high glucose, fatty acids, and oxidative attacks are currently highlighted as the main stressors. However, published studies typically describe one single or combined stress conditions. The present study compared these different stressors individually in the same experimental set, revealing specific molecular targets of cell injuries. This approach identified mitochondrial stress-specific signatures.

## Acknowledgments

We are grateful to Gaelle Chaffard, Andrea Rotmistrovsky Valcarcel, Deborah Strebel and Didier Chollet (University of Geneva) for excellent technical assistance. We thank Amos Bairoch, Alexandre Masselot and Catherine Zwahlen (Swiss Institute of Bioinformatics, Geneva) for their help with bioinformatics and Cytoscape representation.

## Supporting Information

Figure S1 **Secretory responses of INS-1E cells after stress exposure.** INS-1E cells were exposed for 3 days to different culture conditions: 5.6 mM glucose (G5.6), 25 mM glucose (G25), 0.4 mM palmitate (Palm), 0.4 mM oleate (Olea). Culture at 11.1 mM glucose (G11) served as no stress (negative control) and transient oxidative stress at day 0 (200 µM H_2_O_2_ for 10 min) served as acute stress (positive control). At day 3, cells were washed and insulin secretion was measured at basal 2.5 mM (Basal, white bars) and stimulatory 15 mM glucose concentrations (Stimul., black bars) following a 30 min incubation period. Values are means ± SEM of 6 independent experiments, each done in duplicate. *P<0.05, **P<0.01, ***P<0.005 *versus* corresponding G11 controls; # *P*<0.05, ## *P*<0.01, ### *P*<0.005 *versus* corresponding basal secretions.
(TIF)

Figure S2 **Expression profile of various carriers of the Slc25 gene family in INS-1E cells cultured 3 days under different stress conditions.** Transcript levels in INS-1E cells cultured without stress at 11.1 mM glucose concentration (G11, control) or exposed to different experimental conditions as described in Methods. Transcript levels were normalized to those of 18S. The relative quantification of the genes of interest is given as mRNA levels normalized to the control value of G11. Results are means ± SEM of 2 independent experiments done in triplicate. *P<0.05, **P<0.01, ***P<0.005 versus G11 controls.
(TIF)

Figure S3 **Transcriptome and proteome from INS-1E cells cultured 3 days after transient oxidative stress.** The schemes provide a global view of the expression of the 60 genes at transcript (node core) and protein (node border) levels. The expressed genes were grouped using the Cytoscape software according to their protein subcellular localization (from the databases UniProtKB/SwissProt and neXtProt); plasma membrane (PM), cytoplasm, nucleus, mitochondrial inner membrane, matrix, endoplasmic reticulum (ER), and peroxisome. Node shape: rectangles represent transporters or receptors, circles are enzymes or stress proteins, octagons show energy related sensors, round rectangles transcription factors, and hexagons carriers. Colors reflect changes in expression levels versus G11 controls: green and red for significant (P<0.05) down- and upregulation, respectively. Dark green: levels<0.5; light green: levels >0.5 but <0.8; pink: levels >1.2 but <1.5; red: levels >1.5. Border colors: black no change in protein level; grey not tested.
(TIF)
