# Supplementary figures and images for: Changes in Mitochondrial Carriers Exhibit Stress-Specific Signatures in INS-1Eβ-Cells Exposed to Glucose Versus Fatty Acids
Source: PLoS One. 2013 Dec 12;8(12):e82364. doi: 10.1371/journal.pone.0082364 (PMC3861392; doi:10.1371/journal.pone.0082364)

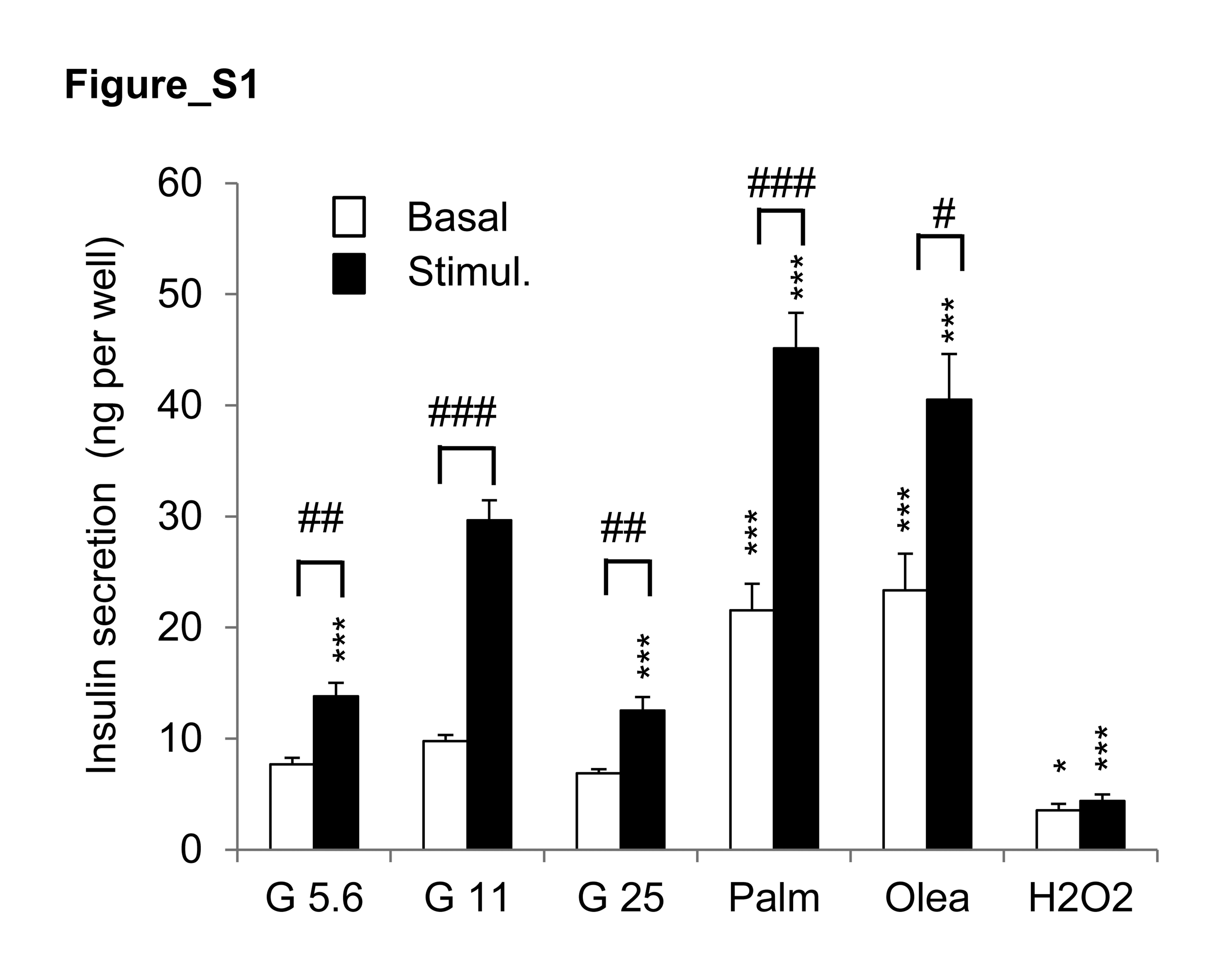

Supplement: Figure S1 — Secretory responses of INS-1E cells after stress exposure. INS-1E cells were exposed for 3 days to different culture conditions: 5.6 mM glucose (G5.6), 25 mM glucose (G25), 0.4 mM palmitate (Palm), 0.4 mM oleate (Olea). Culture at 11.1 mM glucose (G11) served as no stress (negative control) and transient oxidative stress at day 0 (200 µM H2O2 for 10 min) served as acute stress (positive control). At day 3, cells were washed and insulin secretion was measured at basal 2.5 mM (Basal, white bars) and stimulatory 15 mM glucose concentrations (Stimul., black bars) following a 30 min incubation period. Values are means ± SEM of 6 independent experiments, each done in duplicate. *P<0.05, **P<0.01, ***P<0.005 versus corresponding G11 controls; # P<0.05, ## P<0.01, ### P<0.005 versus corresponding basal secretions. (TIF) [file pone.0082364.s001.tif]

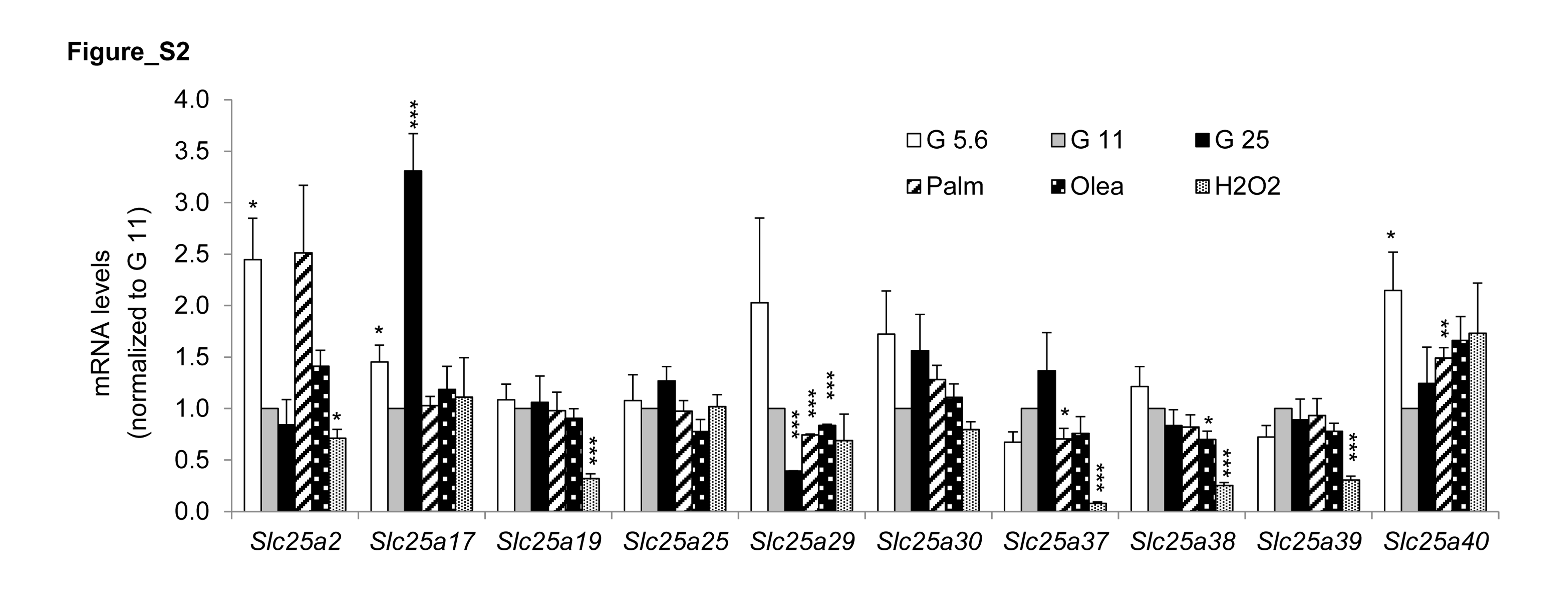

Supplement: Figure S2 — Expression profile of various carriers of the Slc25 gene family in INS-1E cells cultured 3 days under different stress conditions. Transcript levels in INS-1E cells cultured without stress at 11.1 mM glucose concentration (G11, control) or exposed to different experimental conditions as described in Methods. Transcript levels were normalized to those of 18S. The relative quantification of the genes of interest is given as mRNA levels normalized to the control value of G11. Results are means ± SEM of 2 independent experiments done in triplicate. *P<0.05, **P<0.01, ***P<0.005 versus G11 controls. (TIF) [file pone.0082364.s002.tif]

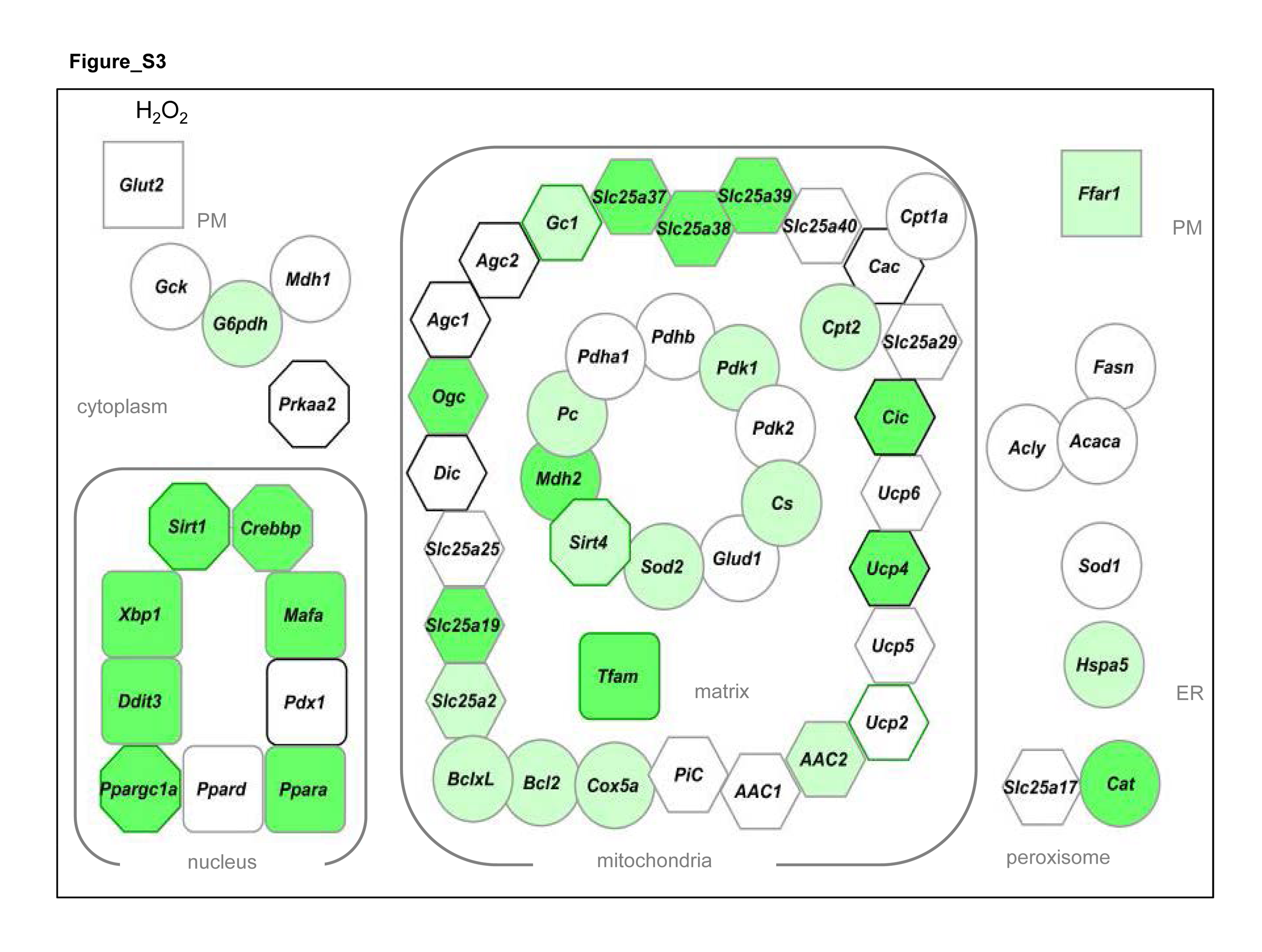

Supplement: Figure S3 — Transcriptome and proteome from INS-1E cells cultured 3 days after transient oxidative stress. The schemes provide a global view of the expression of the 60 genes at transcript (node core) and protein (node border) levels. The expressed genes were grouped using the Cytoscape software according to their protein subcellular localization (from the databases UniProtKB/SwissProt and neXtProt); plasma membrane (PM), cytoplasm, nucleus, mitochondrial inner membrane, matrix, endoplasmic reticulum (ER), and peroxisome. Node shape: rectangles represent transporters or receptors, circles are enzymes or stress proteins, octagons show energy related sensors, round rectangles transcription factors, and hexagons carriers. Colors reflect changes in expression levels versus G11 controls: green and red for significant (P<0.05) down- and upregulation, respectively. Dark green: levels<0.5; light green: levels >0.5 but <0.8; pink: levels >1.2 but <1.5; red: levels >1.5. Border colors: black no change in protein level; grey not tested. (TIF) [file pone.0082364.s003.tif]
